# Clinicopathological Features, Treatment Response, and Outcome of Rosai‐Dorfman Disease in Two Children

**DOI:** 10.1002/ccr3.72471

**Published:** 2026-04-19

**Authors:** George Evele, Kouya Francine, Richard Bardin

**Affiliations:** ^1^ Paediatric Oncology Unit/Mbingo Baptist Hospital Mbingo Cameroon; ^2^ Pathology Unit/Mbingo Baptist Hospital Mbingo Cameroon

**Keywords:** cervical lymphadenopathy, complete remission, emperipolesis, histiocytic disorder, rare, Rosai‐Dorfman disease

## Abstract

Rosai‐Dorfman disease is a rare non‐Langerhans cell histiocytic disorder. It is common in male children and young adults of African descent. It is classified into sporadic and familial types. The most common clinical presentation is massive bilateral cervical lymphadenopathy associated with constitutional symptoms. Histiocytic emperipolesis is a diagnostic hallmark for Rosai‐Dorfman disease. The diagnostic criteria for Rosai‐Dorfman disease are large histiocytic cells that stain positive for CD68 and S100, but negative for CD1a. We present two cases of sporadic Rosai‐Dorfman disease in two males of African descent. The first patient had classic nodal Rosai‐Dorfman disease; however, he was initially misdiagnosed as lymphoma. He responded poorly to cytotoxic drugs, but achieved a durable complete remission with three cycles of oral prednisolone. The second case had both nodal and extranodal Rosai‐Dorfman disease. He initially responded well to steroids, but the response was short‐lived. However, he achieved complete remission with a combination of oral methotrexate and vincristine. Rosai‐Dorfman disease usually has a simple and uncomplicated clinical course. There is no universal standard guideline for managing Rosai‐Dorfman disease. However, patients with symptomatic disease, those experiencing emergency symptoms, and cases of relapse will require medical or surgical intervention to improve their outcomes.

## Key Clinical Message

Rosai‐Dorfman disease may be misdiagnosed as tuberculous adenitis or lymphoma due to similarities in clinical presentation. Histiocytic emperipolesis is a diagnostic hallmark for Rosai‐Dorfman disease. Nonetheless, the definitive diagnosis of Rosai‐Dorfman disease involves identifying histiocytic cells that stain positive for CD68 and S100 but negative for CD1a.

## 1 Introduction

Rosai‐Dorfman disease (RDD) is a rare and distinct form of non‐Langerhans cell histiocytosis [1]. RDD is characterized by the abnormal accumulation or infiltration of activated tissue‐resident macrophages (histiocytes), resulting in a range of systemic manifestations [1, 2].

In 1965, Destombes was the first to report histopathological findings of RDD in four African children with lymphadenopathy, a condition he termed “Adenitis with Lipid Excess.” [2]. Subsequently, in 1969, Juan Rosai and Ronald Dorfman characterized “Adenitis with Lipid Excess” in four patients who presented with massive cervical lymphadenopathy, coining the term “Sinus histiocytosis with massive lymphadenopathy (SHML)” [2].

In 2016, the Histiocytic Society classified RDD as belonging to the R group, with two subtypes: familial and sporadic [1]. The sporadic subtype is subcategorized into the classical nodal disease, extranodal, RDD associated with neoplasia or immune disease, and the unclassified [1].

RDD has a prevalence of 1 in 200,000, predominantly affecting children and young adults of African descent [2, 3]. In Africa, RDD is commonly diagnosed in children aged 10 years and younger, with a slight preponderance for males over females [4].

The etiology of RDD is not well understood [1, 2]. RDD is thought to stem from a dysfunctional immune response following viral infections like Epstein–Barr virus and HIV, though this is still unconfirmed by studies [2]. RDD exhibits a variety of phenotypic features, which include both benign and neoplastic characteristics [1, 2, 5]. The detection of pathogenic somatic mutations in genes of the mitogen‐activated protein kinase/extracellular signal‐regulated kinase (MAPK/ERK) pathway indicates that a subset of patients with RDD may have a clonal neoplastic origin of the disease [5, 6]. There is no gold standard for RDD management, but Abla et al. provided a consensus guide for its diagnosis and treatment [2].

We present two cases of sporadic Rosai‐Dorfman disease: one with nodal involvement and the other with both nodal and extranodal disease.

## 2 Case Presentation

### 2.1 Case 1

A 9‐year‐old male was referred to our pediatric oncology unit in September 2023, with a history of progressive bilateral painless neck swellings for 3 months. This was associated with intermittent low‐grade fever. There was no history of night sweats, weight loss, cough, or anorexia. He had no previous history of cancer and no known exposure to tuberculosis. On clinical examination, he had a bilateral, asymmetric enlargement of the cervical lymph nodes, with the largest measuring approximately 10 cm × 6 cm on the left and around 4 cm × 4 cm on the right. The lymph nodes were rubbery, mobile, with no change in overlying skin, not fixed, and mildly tender. The rest of the exam was normal. The presumptive diagnosis was Hodgkin lymphoma with a differential of tuberculous cervical lymphadenitis.

### 2.2 Investigations and Presumptive Treatment

The initial laboratory investigations included a complete blood count, which revealed a leucocytosis of 19,070 cells/μL, neutrophilia of 15,367 cells/μL, moderate microcytic hypochromic anemia at 9.8 g/dL, monocytosis of 2566 cells/μL, and a normal platelet count. The HIV serology and malaria parasite tests were negative. The chest X‐ray and abdominal ultrasound were benign.

**FIGURE 1:**
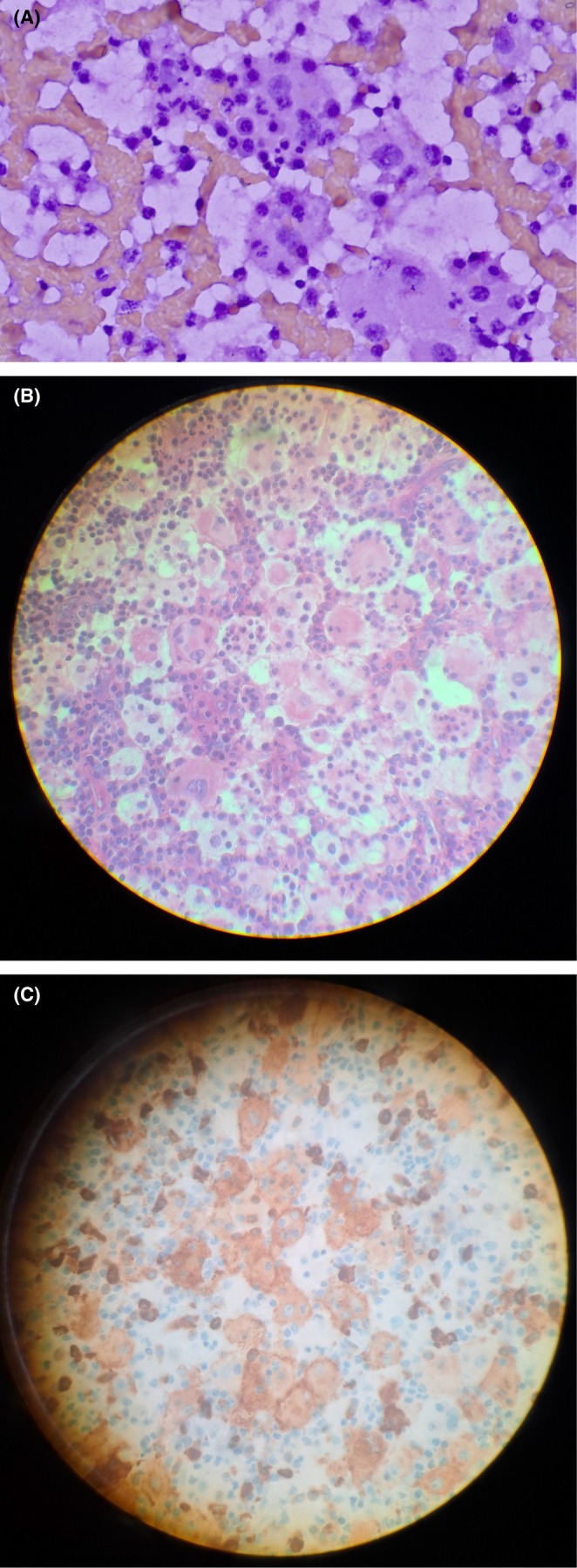
(A) (Case 1): Cytopathology of cervical lymph node on hematoxylin and eosin shows histiocytoid cells with emperipolesis. (B) (Case 1) H and E stain of cervical lymph node after excisional lymph node biopsy (40×): Histiocytic cells with watery‐clear cytoplasm and small lymphocytes within their cytoplasm. (C) (Case 1) CD163 positive histiocytic cells stain brown (40×).

The first fine‐needle aspiration (FNA) of the left cervical node was performed. The cytopathology analysis revealed a mixed lymphoid population with small lymphocytes as the majority. Reed‐Sternberg cells or other evidence of malignancy were not seen. An incisional biopsy was performed on the left cervical lymph node, and the histopathologic analysis showed monomorphic small round cell proliferation. The findings did not show any signs of necrotizing granulomatous inflammation that would suggest the presence of *Mycobacterium tuberculosis* . A second FNA of the largest cervical lymph node was performed, and the findings included many vascularized cellular fragments and abundant large round cells with foamy cytoplasm, often marked nuclear atypia, containing other cells such as neutrophils and plasma cells. Some large cells had a morphology resembling Reed‐Sternberg cells. The conclusion was histiocytoid proliferation with atypia associated with either hemophagocytosis or emperipolesis (Figure 1A). An excisional biopsy of the predominant left cervical lymph node was done.

An initial diagnosis of stage II Hodgkin lymphoma was made based on the persistent bilateral lymphadenopathy and cells with a semblance to Reed‐Sternberg cells on cytopathology. To reduce the risk of airway compromise, the patient received an initial chemotherapy regimen of cyclophosphamide, vincristine, and prednisolone. One week later, the bilateral neck swellings had reduced by about 40%. As a result, the COMP protocol (cyclophosphamide, vincristine, methotrexate, and prednisolone) was then initiated, and the patient received days 1 and 8. There was a brief clinical reduction in the neck swellings of about 70%, but by the next chemotherapy cycle, they had almost returned to their original sizes. A resistant disease was suspected, and the ABVD (Adriamycin, Bleomycin, Vinblastine, and Dacarbazine) regimen for Hodgkin lymphoma was initiated. Chemotherapy was given on Day 1, with minimal clinical response. Day 15 treatment was not administered because of the initial poor response.

The final histopathological analysis of the excised cervical lymph node revealed a mixed population that includes small, medium, and large lymphoid cells, large histiocytoid cells containing other cells, including plasma cells, lymphocytes, and neutrophils. A large histiocytoid cell with nuclear atypia, including marked nucleomegaly, pleomorphism, and prominent nucleoli. Many of the small and medium‐sized lymphoid cells showed moderate nuclear atypia as well. There was a nodular or follicular appearance with aggregates of large histiocytoid cells exhibiting emperipolesis (Figure 1B). There were fibrous bands. The overall architecture of the lymph node was intact, with massive sinus histiocytosis. Classic Reed‐Sternberg cells were not seen. An immunohistochemical analysis of the specimen was conducted overseas. The positive stains were cluster of differentiation (CD)163 (Figure 1C) and CD15; meanwhile, the negative stains were CD3, CD20, and CD30. The findings were consistent with massive sinus histiocytosis. In this light, Hodgkin and non‐Hodgkin lymphomas were less likely. Other immunohistochemical analyses for S100, CD1a, CD207 (Langerin), and flow cytometry were not performed to exclude other histiocytic disorders. These services are not available in our pathology laboratory. A request was made to the laboratory overseas, but the specimen was inadequate.

### 2.3 Treatment and Outcome

The patient received three cycles of prednisolone at 1 mg/kg/day, tapered over four weeks, in conjunction with weekly cotrimoxazole and a daily calcium‐vitamin D3 supplement. After the third cycle of prednisolone, there was no cervical lymphadenopathy.

### 2.4 Follow‐Up

The patient has been in complete remission for 2 years, with no major adverse events.

## 3 Case 2

A 4‐year‐old male presented as a referral to our pediatric oncology unit in June 2024 with a 2‐month history of painless bilateral neck swellings associated with fever, weight loss, night sweats, and snoring. He had no cough and no exposure to tuberculosis. He underwent multiple courses of oral antibiotics for a suspected upper respiratory tract infection; however, the symptoms continued, leading to a referral. He had no history of cancer. On clinical examination, the conjunctivae were pale, and he had bilateral enlarged cervical lymph nodes. In the right cervical region, the largest lymph node measured approximately 8 cm × 7 cm, and on the left, it was approximately 7 cm × 5 cm. They were rubbery, mobile, and non‐tender, with normal overlying skin. He had bilateral grade 3 non‐inflamed tonsils. The mid‐upper arm circumference was 12.0 cm, and the rest of the exam was benign. The provisional diagnosis was cervical lymphadenitis due to tuberculosis, characterized by lymphadenopathy and constitutional symptoms, with a differential diagnosis of non‐Hodgkin lymphoma.

### 3.1 Investigations

**FIGURE 2:**
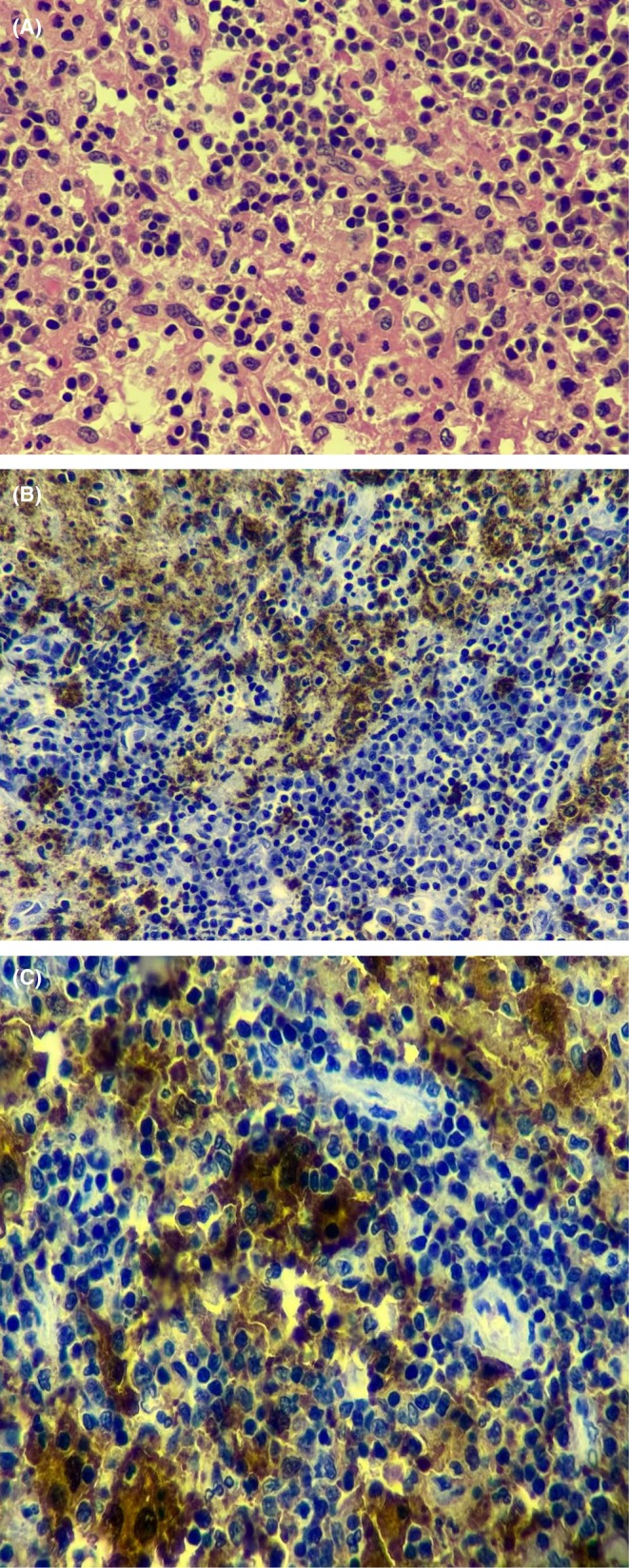
(A) (Case 2): Large histiocytic cells with some harboring lymphocytes on H and E stain (40×). (B) (Case 2): Positive CD68 histiocytic cells in the cervical lymph node. (C) The histiocytic cells stained strongly positive with S100.

The initial complete blood count revealed a leukocytosis of 16,650 cells/μL, neutrophilia of 13,874 cells/μL, moderate microcytic hypochromic anemia of 8.1 g/dL, and a normal platelet count. The complete metabolic panel showed elevated alkaline phosphatase at 293 U/L, mild hypoalbuminemia at 3.18 g/dL, mild hyponatremia, mild hypochloremia, and the rest within normal ranges. HIV serology was negative, and the C‐reactive protein (CRP) was 161 mg/L. Enlarged lymph nodes were observed on an abdominal ultrasound near the porta hepatis, with the largest measuring 2.9 cm × 2.5 cm × 2.9 cm. An FNA of the cervical lymph node was negative for GeneXpert. The cytopathology analysis of the right cervical lymph node revealed a mixed lymphoid population with small lymphocytes as the majority. Numerous large histiocytes were seen containing intact cells, predominantly small lymphocytes and neutrophils. Malignant cells were not identified. An excisional biopsy of a cervical lymph node was done, and the histopathologic analysis revealed extensive sinus histiocytosis with numerous large cells containing lymphocytes (Figure 2A). The immunohistochemical analysis showed CD68 (Figure 2B) and S100 (Figure 2C) were strongly positive within the sinus histiocytic cells, while a stain for CD1a was negative. These findings were consistent with Rosai‐Dorfman disease presenting as classic nodal and extranodal disease.

### 3.2 Treatment and Outcome

He received intravenous dexamethasone at a tapered dose of 0.5 mg/kg daily for 1 week with an excellent reduction of nodal sizes within the first 48 h. The initial response after 1 week was excellent, with more than 70% reduction in nodal sizes and complete resolution of the bilateral tonsillar enlargement. To minimize the long‐term adverse effects of dexamethasone, prednisolone, a less potent substitute, was chosen. He continued with prednisolone at a tapered dose of 1 mg/kg over 4 weeks, along with weekly cotrimoxazole and calcium‐vitamin D tablets.

However, he developed a relapse of fever, night sweats, and weight loss, but stable lymph node sizes after 3 weeks of steroids. A possible steroid resistance was suspected. The treatment was adjusted to weekly oral methotrexate 20 mg/m^2^ and vincristine 1.5 mg/m^2^ for 2 months. By the end of the treatment, there was about a 90% clinical reduction of the bilateral neck swellings. The fever and night sweats subsided immediately after starting the combination therapy, and his weight steadily improved. He was then placed on monthly surveillance, and by the end of 6 months, the enlarged lymph nodes had completely resolved.

### 3.3 Follow‐Up

The patient has been in complete remission for more than a year now with no significant adverse event noted.

## 4 Discussion

RDD is a systemic disease with a spectrum of clinical features depending on the affected organ system [2]. The most prevalent clinical feature of RDD is bilateral, painless, massive cervical lymphadenopathy, sometimes associated with constitutional symptoms [1]. This concurs with both cases presented. However, extranodal RDD occurs in about 43% of patients [2]. Case one was diagnosed with classic nodal RDD, while case two had both nodal and extranodal disease due to the bilateral grade 3 tonsillar enlargement, which was responsive to treatment [2]. Classic RDD with bilateral cervical lymphadenopathy associated with fever, fatigue, weight loss, and night sweats can be misdiagnosed as cervical tuberculous adenitis (scrofula) [4]. *Mycobacterium tuberculosis* (MTB) is the leading cause of persistent cervical lymphadenopathy and a significant cause of morbidity and mortality in children in low‐ and middle‐income countries [7]. Fine‐needle aspiration (FNA) is a simple procedure used to collect samples for the GeneXpert test, which has a sensitivity of approximately 86% to 96% in diagnosing MTB [7]. Case two had a negative GeneXpert result from an FNA of a cervical lymph node, while case one was not tested. Laudin et al. [4] found that about 11% of Rosai‐Dorfman disease cases in Africa were misdiagnosed as tuberculosis (TB) and treated with anti‐TB drugs. Sporadic RDD is more common in children and young adults, particularly among males of African descent, and these concur with our cases [1]. In Africa, most reported cases of RDD are found in young children, particularly those aged 10 and under, aligning with the two presented cases [4]. Classic RDD symptoms usually develop gradually over 3–6 months and can sometimes be misdiagnosed as hematolymphoid neoplasm [8]. The first case was incorrectly diagnosed as Hodgkin lymphoma.

Hodgkin lymphoma (HL) is a unique type of B‐cell‐derived lymphoid neoplasm with distinct clinical and histological characteristics [9]. It is common in adolescents but rare in those under five [2, 9]. The clinical presentation typically involves a slow‐growing, painless lymphadenopathy, which may or may not be associated with constitutional symptoms [9]. The cervical lymph nodes are the most commonly affected [9]. The histopathological hallmark of HL is the presence of large Reed‐Sternberg cells, which are multinucleated or mononuclear Hodgkin cells with conspicuous nucleoli [9]. These cells stain positive for CD30 and express CD20 only in rare cases [9]. On the other hand, non‐Hodgkin lymphoma is a heterogeneous group of malignant B‐cell or T‐cell lymphoproliferative neoplasms characterized by the absence of Reed‐Sternberg cells [10]. It is a common childhood malignancy [10]. They can present with nodal or extranodal involvement associated with constitutional symptoms [2, 10]. CD20 is a commonly used marker for B‐cell tumors, while CD3 is used for T‐cells [10]. The clinical presentations are similar to those of RDD, which can lead to misdiagnosis [2]. Also, Langerhans cell histiocytosis (LCH) is a common differential to RDD [2]. It is a rare histiocytic disorder classified in the L‐group [1]. It is characterized by tissue infiltration by Langerhans cell histiocytes, and can affect any organ system [1]. The bones of children are most affected; lymph nodes are involved in approximately 5%–10% of cases [1]. The definitive diagnosis of LCH is histiocytes that stain positive for CD1a and CD207 [1, 2].

Massive lymphadenopathy is generally characterized by an enlarged lymph node measuring 7 cm or more in diameter [11]. Not all RDD patients have massive lymphadenopathy; some have smaller nodes, and extranodal cases may lack lymphadenopathy entirely [2, 11]. The cases presented had massive lymphadenopathy. Diagnosing RDD through histopathology can be challenging and often necessitates multiple biopsy samples for confirmation [11]. *Goyal* et al. [11] reported that the median number of biopsies required for RDD diagnosis was two, with a range of one to six. The cases presented had multiple biopsies. Diagnostic delays in RDD patients burden finances and health, especially those misdiagnosed as neoplasms and treated with cytotoxic drugs [4, 11].

The histopathological features of Classic RDD show sinusoids infiltrated with numerous large histiocytic cells [2]. These cells have round or oval hypochromatic nuclei, “watery‐clear” cytoplasm, and a prominent nucleolus [2, 12]. Additional characteristics of histiocytic cells include multiple nuclei, nuclear atypia, and rare mitotic figures [12]. Some of these features mimic those of neoplasms [12]. Histiocytic emperipolesis is a hallmark of RDD, characterized by lymphocytes and other cells within the cytoplasm of histiocytes [1, 2]. Emperipolesis is a cell‐in‐cell biological phenomenon where a cell invades another and lives in the host cell's cytoplasm without being destroyed [1, 2]. It is significant for diagnosing RDD; however, due to its variability among patients, it is not a definitive diagnostic feature [2]. The definitive diagnostic features of RDD include the immunophenotypic characteristics of large histiocytes that are positive for CD68 and S100, but negative for CD1a or CD207 (Langerin) [2]. Other positive stains in RDD are CD14 and CD163 [1]. The second patient fulfilled the diagnostic criteria for RDD, but the first case only had a positive CD163 test. CD1a staining was not performed for case one to exclude Langerhans cell histiocytosis, which is a differential diagnosis of RDD. But in the first patient, other significant stains were negative for T‐cell lymphoma (CD3), B‐cell lymphoma (CD20), and Hodgkin lymphoma (CD30). CD15 is not a diagnostic marker for RDD; however, a small proportion of tissue‐resident macrophages can express it [1, 12]. Since CD30 was negative, which is more definitive than CD15 for diagnosing Hodgkin lymphoma, CD15 positivity was not considered diagnostic [12]. In both cases, RDD was not considered to be associated with neoplasms, and the most commonly reported RDD‐associated neoplasm is lymphoma [2]. Our laboratory currently lacks the capacity to test for driver mutations in the cases presented. Some RDD patients have significant MAPK pathway mutations that may inform treatment. RDD patients may show elevated inflammatory markers, leukocytosis with neutrophilia, and normocytic hypochromic anemia [2].

There is no universally accepted gold standard for managing RDD [2]. In 2018, *Abla* et al. [2] published consensus recommendations for the clinical diagnosis and management of RDD. In about 50% of cases, RDD follows a benign course and may resolve spontaneously without treatment [13]. Observation is indicated for RDD cases with uncomplicated peripheral lymphadenopathy [2]. Other management options include corticosteroids, surgery, radiotherapy, immunotherapies, and tyrosine kinase inhibitors [2, 11]. These are primarily indicated for patients with symptomatic disease, relapses, refractory disease, and those with emergency symptoms [2]. The first patient was initially misdiagnosed with Hodgkin lymphoma and received two different chemotherapy regimens with short‐lived responses. He had short courses of corticosteroid during the first regimen, but the response was momentary. He achieved complete remission of all symptoms with a durable response by using a tapering dose of prednisone at 1 mg/kg/day, repeated in a four‐week cycle over 3 months. The second patient had a partial response to corticosteroids and a complete response when a combination of low‐dose methotrexate and vincristine was administered [2]. *Abla* et al. [2] recommended the use of vincristine or oral methotrexate as single or combined therapy for those who relapsed while on corticosteroids. A relapse rate of about 50% has been reported with the use of corticosteroids in RDD patients [11]. While some clinicians prefer to increase the dose of corticosteroid for RDD cases with relapse, we declined to augment the corticosteroid dose for the second case to minimize the adverse events [11]. Systemic corticosteroids play key roles as anti‐inflammatory agents, immunomodulators, and antineoplastic medications [14]. However, their adverse events can be life‐threatening [15]. Some metabolic adverse effects linked to corticosteroid use include hypertension, hyperglycemia, type 2 diabetes, weight gain, hyperlipidemia, osteoporosis, and adrenal suppression [14, 15]. Additional adverse effects consist of increased risk of infection, gastrointestinal bleeding, impaired wound healing, and psychiatric disturbances [14]. The likelihood of experiencing negative side effects can increase with both the dosage and the duration of use [14, 15]. Some guidelines recommend the use of antibiotics, tapering doses, the use of calcium, and vitamin D to minimize the adverse effects of long‐term use of corticosteroids [14, 15].

The clinical outcomes for the majority of patients with Rosai‐Dorfman disease (RDD) are favorable [1, 2]. However, mortality and morbidity rates are high among patients with multifocal disease, extranodal disease, and refractory disease [2]. In particular, mortality can exceed 40% for those with RDD of the kidneys or lungs [2].

## 5 Conclusion

Rosai‐Dorfman Disease is a rare non‐Langerhans cell histiocytic disorder with symptoms that mimic those of lymphoma and tuberculosis. The clinical course is generally benign for asymptomatic RDD cases. Patients with symptomatic disease may require medical or surgical management to improve outcomes. The cases discussed are the first from our institution. We highlight the necessity of raising awareness about the clinical presentation, diagnostic criteria, and management challenges associated with symptomatic RDD disease cases. This presentation aims to enhance understanding and awareness, ultimately reducing the misdiagnosis of RDD in areas where *Mycobacterium tuberculosis* (MTB) and lymphomas are common.

## Author Contributions

**George Evele:** conceptualization, data curation, investigation, methodology, supervision, visualization, writing – original draft, writing – review and editing. **Kouya Francine:** methodology, writing – review and editing. **Richard Bardin:** investigation, supervision, validation, writing – review and editing.

## Funding

The authors have nothing to report.

## Consent

The parents of both patients gave written informed consent for the study and images for this publication.

## Conflicts of Interest

The authors declare no conflicts of interest.

## Data Availability

The consent forms are available upon request.
